# Case Report: Simultaneous chronic lymphocytic leukemia and macrofocal multiple myeloma with extramedullary plasmacytoma

**DOI:** 10.3389/fonc.2026.1747723

**Published:** 2026-01-28

**Authors:** Yingming Jin, Shuyan Wang, Zhi Fang, Suying Qian

**Affiliations:** 1 Department of Hematology and Oncology, Ningbo No.2 Hospital, Ningbo, China; 2 Department of Histopathology, Ningbo Clinical Pathological Diagnosis Center, Ningbo, China

**Keywords:** chronic lymphocytic leukemia, extramedullary plasmacytoma, macrofocal multiple myeloma, supraclavicular lymph node, thoracic vertebra

## Abstract

There are extremely few reports of concurrent multiple myeloma (MM) and chronic lymphocytic leukemia (CLL) in the literature. To the best of our knowledge, the existence of concurrent CLL and macrofocal MM (MFMM), presenting as an extramedullary plasmacytoma (EMP) in a lymph node, has not been previously documented. Herein, we report a case of a 67-year-old male presenting with backache persisting for more than 1 month. Laboratory test results revealed a markedly elevated white blood cell count (57.0 × 10^9^/L) with normal platelet count and hemoglobin level. Positron emission tomography/computed tomography scan showed high fluorodeoxyglucose uptake in the 10^th^ thoracic vertebra (T10), T12, and the left supraclavicular lymph node. Thus, the patient underwent bone marrow aspiration, followed by T10 and left supraclavicular lymph node biopsy. Pathological analysis revealed infiltration by plasmacytoma and neoplastic B-lymphocytes. A final diagnosis of MFMM concurrent with CLL was established. The patient was subsequently treated with bortezomib, lenalidomide, and dexamethasone. After three treatment cycles, a complete response was achieved. However, treatment was discontinued due to grade 3 peripheral sensory neuropathy, and therapy was switched to daratumumab and dexamethasone. The patient remains under outpatient follow-up, with a 1-year follow-up duration.

## Introduction

Chronic lymphocytic leukemia (CLL) is the most prevalent form of adult leukemia in Western countries, characterized by clonal expansion of CD5^+^CD23^+^ B cells in the peripheral blood, bone marrow (BM), and lymphoid tissues. A conclusive diagnosis of CLL requires the presence of sustained lymphocytosis (≥5 × 10^9^/L) for no less than 3 months (1). Many patients do not exhibit symptoms. Symptomatic individuals typically experience weakness, lymphadenopathy, splenomegaly, and infections.

Multiple myeloma (MM) is the second most prevalent type of hematological malignancy, defined by presence of aberrant clonal plasma cells expressing CD38^+^ and CD138^+^ in the BM (2). Macrofocal MM (MFMM) is a rare type of MM, accounting for 3%–5% of all MM cases (3, 4). Its manifestations include clonal plasma cells comprising <20% of the BM, multiple lytic bone lesions, and the absence of anemia, renal insufficiency, and hypercalcemia (3).

MM and CLL are hematological malignancies originating from the B-lymphoid lineage. Although each disease is relatively common individually, the concurrent occurrence of CLL and MM in the same patient is extremely rare. Herein, we present a unique and extremely rare case involving the diagnosis and treatment of a patient with coexisting MFMM and CLL.

## Case report

A 67-year-old man with a medical history of hypertension was admitted to the Department of Hematology and Oncology at our institution on December 11, 2024, due to backache and left lower-quadrant pain persisting for more than 1 month. Laboratory test results revealed a high white blood cell count (57.0 × 10^9^/L), high lymphocyte count (50.7 × 10^9^/L), and normal platelet count (208 × 10^9^/L) and hemoglobin level (145 g/L). Biochemical tests showed normal lactate dehydrogenase (LDH), calcium, and creatinine levels. Routine serum immunoglobulin levels and β2-microglobulin levels were unremarkable (**Table 1**).

**Table 1:** Key laboratory findings.

Laboratory items	Results	Reference range
White blood cell count (×10^9^/L)	57.0	3.5–9.5
Lymphocyte count (×10^9^/L)	50.7	1.1–3.2
Hemoglobin (g/L)	145	130–175
Platelet count (×10^9^/L)	208	125–350
Lactate dehydrogenase (U/L)	185	120–250
Calcium (mmol/L)	2.27	2.11–2.52
Creatinine (μmol/L)	71.6	57.0–111.0
β2-microglobulin (mg/L)	2.77	1.30–3.00
Immunoglobulin G (g/L)	8.35	8.60–17.40
Immunoglobulin A (g/L)	3.35	1.00–4.20
Immunoglobulin M (g/L)	0.35	0.30–2.20
Serum κ free light chain (mg/L)	19.40	3.30–19.40
Serum λ free light chain (mg/L)	1409.38	5.71–26.30
Free light chain ratio (κ/λ)	0.014	0.26–1.65

**Figure 1:**
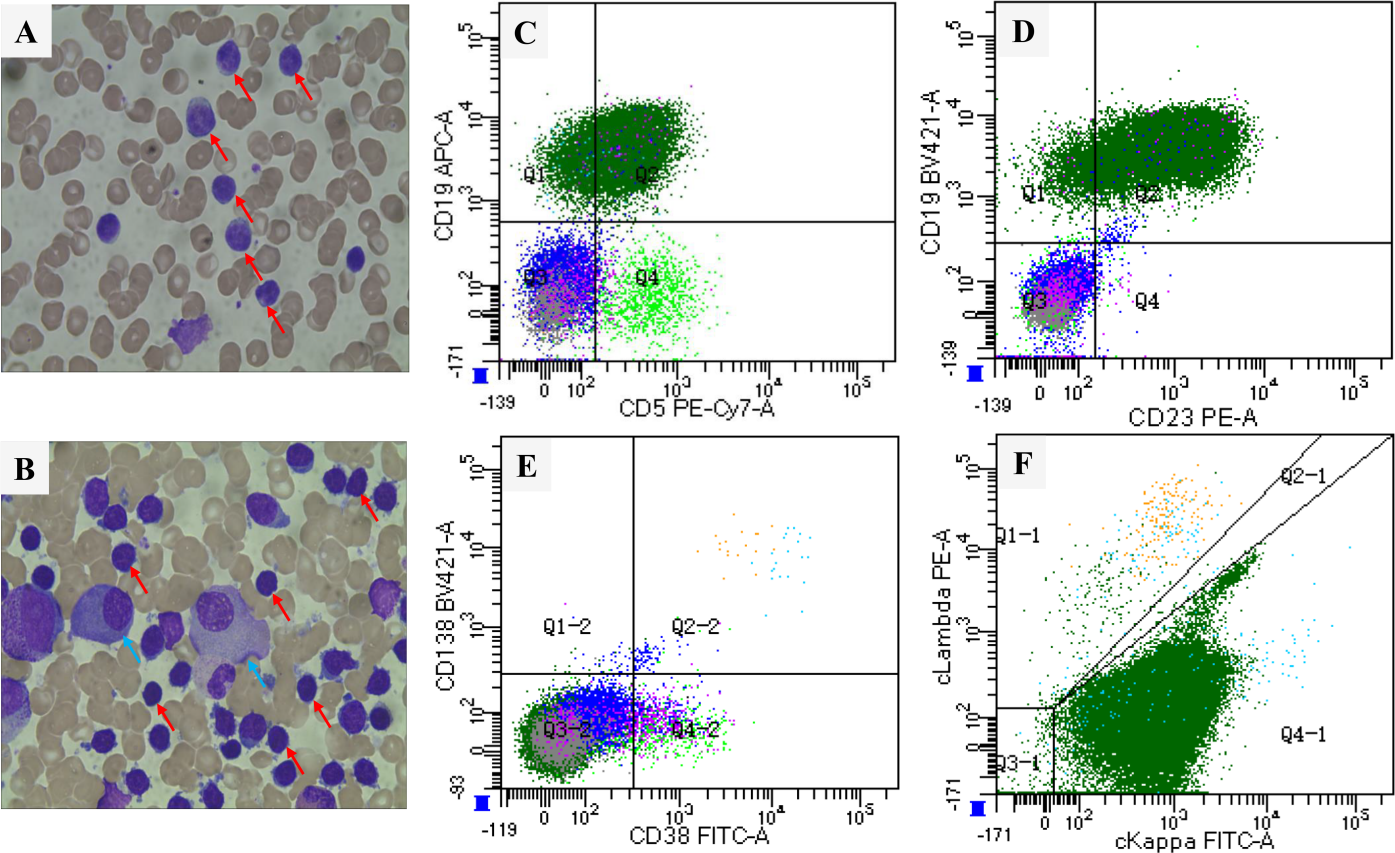
Smear (Wright-Giemsa stain, ×100) showing mature lymphocytes (red arrow) and plasma cells (blue arrow): **(A)** Peripheral blood; **(B)** Bone marrow. Flow cytometric analysis of the bone marrow aspirate revealed a population of CD19^+^ B cells with aberrant expression of CD5 **(C)** and CD23 **(D)**, along with a small subset of neoplastic plasma cells (CD38^+^/CD138^+^) **(E, F)**.

Serological testing revealed active hepatitis B virus (HBV) infection. Peripheral blood smear examination revealed 90% lymphocytosis with the presence of smear cells (**Figure 1A**). Flow cytometric analysis of peripheral blood samples revealed a large monotypic B-cell population comprising 82.0% of total events, expressing CD5^+^, CD23^+^, CD200^+^, ckappa (κ)^+^, CD20^dim^, CD22^dim^, and FMC-7^part^. This population tested negative for CD10, CD38, CD138, lambda (λ), and CD103. Based on these findings, the diagnosis of CLL was confirmed (**Supplementary Figure 1**). BM aspiration showed a hypercellular marrow with 81.5% mature lymphocytes and 1.5% plasma cells, indicating B-cell chronic lymphoproliferative disorder (**Figure 1B**). BM flow cytometry detected a B-lymphocyte population with co-expression of CD19^+^, CD5^+^, and CD23^+^, which was positive for CD20 and cκ but negative for FMC-7, CD38, and λ. Meanwhile, the monoclonal plasma cells accounting for 0.1% of all nuclear cells expressed CD38, CD138, cλ, and CD56, but lacked CD19 expression (**Figures 1C–F**; **Supplementary Figure 2**).

**Figure 2:**
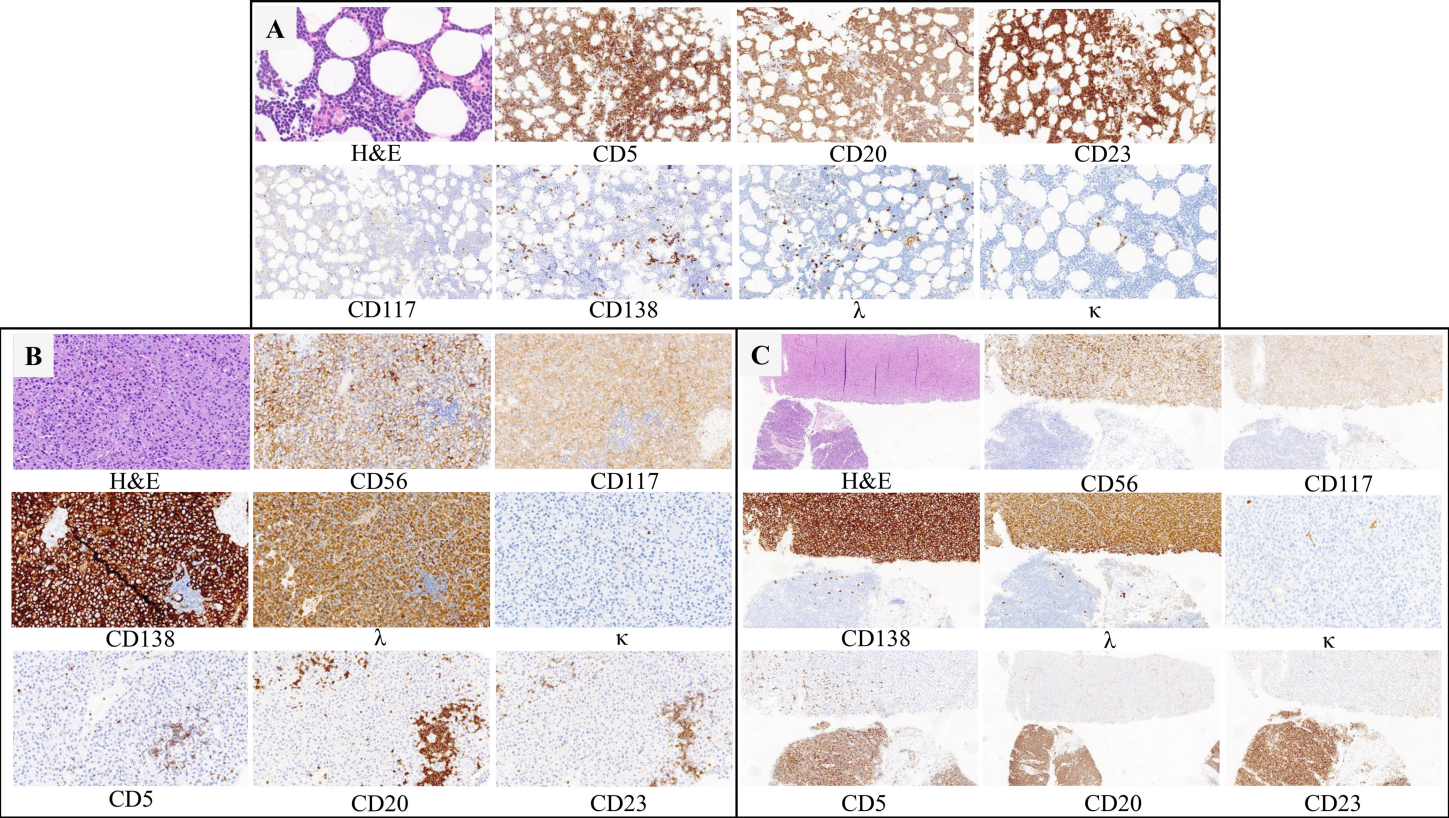
Three sections of biopsies demonstrating features of CLL and PCM. **(A)** Bone marrow (H&E, 40×; IHC,20×) **(B)** T12 (H&E, 20×; IHC,20×). **(C)** The left supraclicular lymph node (H&E, 10×; IHC,10×) H&E-hematoxylin and eosin; IHC-immunohistochemistry.

**Figure 3:**
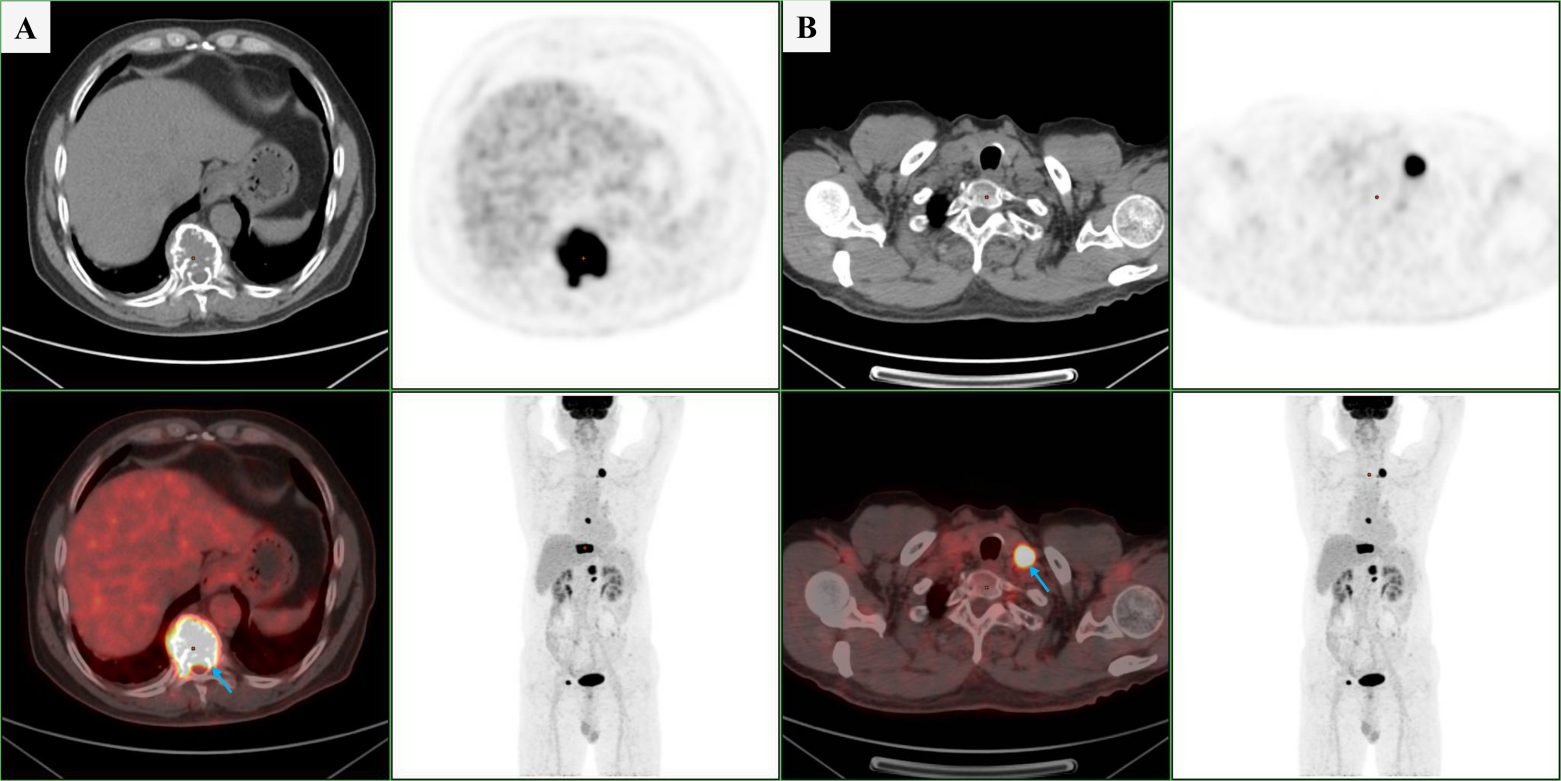
PET/CT demonstrated increased FDG uptake (blue arrow) in the left supraclavicular lymph node **(A)** and the T10 vertebra **(B)**.

Immunohistochemistry of BM biopsy samples showed CD5^+^, CD20^+^, and CD23^+^ with rarely scattered clusters expressing CD138 and λ (**Figure 2A**). The patient was negative for the myeloid differentiation factor 88 leucine 265 to proline (MYD88 L265P) mutation. Positron emission tomography/computed tomography (PET/CT) demonstrated an elevated fluorodeoxyglucose uptake [maximum standardized uptake value (SUVmax): 20.37] in bony structures, particularly in the 10^th^ thoracic vertebra (T10) and T12, with fracture and multiple lytic bone lesions. Hypermetabolic enlarged lymph nodes (SUVmax: 24.56) were observed in the left supraclavicular region and a hypermetabolic nodule beneath the left apex of the pleura (**Figure 3**).

Subsequently, a left supraclavicular lymph node (LN) biopsy was performed on December 13, 2024. Microscopic examination showed disruption of the LN structure with two different areas: one composed of medium-sized cells with a moderate amount of powdery cytoplasm, occasional eccentric nuclei, and a distinct nucleolus, consistent with plasma cell–like morphology and another composed predominantly of small lymphocytes with small cytosol, little cytoplasm, and inconspicuous nucleolus. Immunohistochemistry revealed that the plasma cells were positive for CD38, CD138, λ, and CD56, with a Ki-67 index of 30%. Dispersed clusters of small, mature neoplastic B-lymphoid cells expressing CD5^+^, CD20^+^, and CD23^+^ were also observed, which were negative for cyclin D1 (**Figure 2C**). Due to severe pathological damage and unexplained thoracic vertebral fractures, percutaneous kyphoplasty of the T10 and T12 was performed on December 25, 2024. Postoperative pathological examination showed plasmacytoma infiltration comprising 90%–95% of the thoracic vertebra biopsy cellularity, with B-lymphocytic neoplastic infiltration accounting for the remaining 5%–10%. The plasmacytomas exhibited aberrant expression of CD38, CD138, λ, CD56, and CD117, with a Ki-67 level of 30%. Meanwhile, a low percentage of mature neoplastic B-lymphoid cells tested positive for immunohistochemical stains, including CD5, CD20, and CD23, with a Ki-67 index of 5%–10%. These abnormal B-lymphoid aggregates comprised <10% of the biopsy cellularity (**Figure 2B**). Based on further analysis, serum immunofixation electrophoresis revealed that the monoclonal (M) immunoglobulin was IgA-λ, and protein electrophoresis showed that the serum M protein level was 2.04 g/L. The free light chain levels of λ and κ were 1409.38 and 19.40 mg/L, respectively (normal ranges: 5.71–26.30 vs. 3.30–19.40 mg/L). The Fκ/Fλ was 0.014 (normal range: 0.26–1.65). Cytogenetics revealed 46, X, -Y, inv (1) (p13q42), +mar [8]/46, XY [3]. Fluorescent *in situ* hybridization analyses of purified CD138+ cells was negative for IGH/MAF, IGH/FGFR3, IGH/CCND1, RB1, D13S319, 1q21, and P53. Thus, the final diagnosis of CLL (Binet stage B; Rai stage I) with concomitant MFMM (Durie–Salmon stage III subgroup A; International Stage System [ISS] stage I; revised ISS [R-ISS] stage I; second revised ISS [R2-ISS] stage I) was achieved. Subsequently, the patient was treated with bortezomib, lenalidomide, and dexamethasone (VRd) along with monthly bisphosphonate therapy. He also received daily entecavir for chronic HBV infection and was monitored for CLL. After three cycles of VRd therapy, the patient achieved complete response according the International Myeloma Working Group (IMWG) criteria. However, treatment was discontinued due to grade 3 peripheral sensory neuropathy, and therapy was switched to daratumumab and dexamethasone. The patient is currently under outpatient follow-up.

## Discussion

CLL and MM are chronic B-cell neoplasms representing distinct stages of B-cell maturation. Their coexistence is extremely rare, and simultaneous diagnosis of both conditions is even less common (5–7). MM can be readily identified when CLL is accompanied by clonal BM plasma cells >10%. However, cases of CLL with BM plasma cells <10% or plasmacytoid differentiation can be easily misdiagnosed or overlooked, which makes rapid diagnosis challenging. Differential diagnoses including marginal zone lymphoma (MZL), Waldenström macroglobulinemia (WM)/lymphoplasmacytic lymphoma (LPL), and monoclonal gammopathy of undetermined significance (MGUS) should be ruled out. CD5 and CD23 positivity is crucial for distinguishing CLL from other indolent lymphomas and represents the most important diagnostic feature of CLL/small lymphocytic lymphoma (SLL) (8). Regarding WM/LPL, the MYD88 L265P mutation is common and has significant diagnostic value (9). Additionally, the ancillary protein in WM/LPL is typically (though not invariably) IgM. MZL always shows strong surface expression of CD20 and immunoglobulin, whereas CLL typically exhibits weaker CD20 and surface immunoglobulin expression (10). The diagnostic criteria for MGUS include a serum monoclonal protein level of <3 g/dL (typically non-IgM), clonal BM plasma cell level of <10%; and absence of end-organ damage (11). The diagnosis of MM is supported by clinical and radiographic findings, such as osteolytic lesions, hypercalcemia, and frequently a non-IgM paraprotein-features rarely observed in other lymphoid malignancies including LPL, MZL, and CLL (2).

An SUVmax > 10 on PET/CT is a novel and reliable indicator for detecting Richter syndrome (RS), and biopsy of the index lesion is therefore recommended for definitive diagnosis (12). In this context, a potential diagnostic pitfall was the misclassification of the concurrent neoplasm as Richter transformation of CLL. To clarify the diagnosis, the patient underwent PET/CT-guided biopsy. However, a paradoxical finding was observed: BM examination revealed that CLL cells constituted the majority of total cellularity, with a minor proportion of plasmacytomas. In contrast, biopsy results of the LN and thoracic spine showed contradictory patterns. Specifically, the enlarged LN—a common manifestation of CLL but rarely observed in MM—showed extramedullary infiltration by plasmacytoma. Undoubtedly, MFMM accounts for the aforementioned findings. It is a rare malignant clonal plasma cell disorder, accounting for approximately 4% of all MM cases in China (4, 13). Distinct from classic MM, MFMM is characterized by low-level BM plasmacytoma infiltration (<20%), multiple osteolytic lesions, and the absence of typical end-organ damage associated with MM, such as anemia, renal insufficiency, and hypercalcemia. Notably, MFMM is more prone to present with EMP involving the upper respiratory tract, LNs, and other extrapulmonary sites (3, 4). However, the utility of PET/CT in differentiating MFMM from classic MM remains poorly documented. Dou et al. (4) reported the median SUVmax of 9 patients with MFMM and 16 patients with classic MM were 6.1 (range, 4.2–11.5) and 4.2 (range, 2.4–9.7), respectively (p=0.133), indicating that PET/CT had difficulty in distinguishing between the two. Zhang et al. (14) validated the role of baseline PET/CT in clinically suspected EMP, confirming its efficacy in identifying additional lesions, including LN involvement and distant metastases. Conversely, adverse prognostic parameters, such as elevated LDH and β2 microglobulin levels, and high-risk cytogenetics were less common in patients with MFMM than in those with standard MM. Based on this finding, MFMM was a relatively indolent and less aggressive subtype of myeloma (3, 4, 13). Patients with MFMM also were more likely to be male. In terms of both diagnostic criteria and prognostic characteristics, our case was consistent with MFMM, and RS was conclusively excluded as a diagnostic possibility.

MM and CLL share a common B-lymphocyte origin, implying potential epidemiological and clinical similarities. Several potential mechanisms may account for the concurrence of these two mature B-cell malignancies, including a pro-tumorigenic microenvironment, immune dysfunction, and chemotherapy-associated factors. However, the clonal origin of these two disorders—whether they derive from a shared precursor or independent clones—remains a matter of debate. Some researchers propose that they represent distinct clonal proliferations, while others suggest that neoplastic plasma cell colonies may arise from CLL tumor cells (15, 16). In the present case, flow cytometric immunophenotyping of the BM specimen identified a CD5+/CD23+ κ light chain-restricted B-cell population consistent with CLL, as well as a CD19-negative monoclonal CD38+/CD138+ plasma cell population with λ-light-chain restriction, consistent with plasma cell myeloma. Notably, pathological results of the LN clearly revealed two distinct and separate entities, supporting biclonality rather than transformation. Moreover, both IGHV 4–4 and IGHV 3–23 mutation rates were >2%. IGHV 3–23 is regarded as one of the common mutations, particularly in patients with CLL, while IGHV 4–4 is rarely reported. Although an IGHV mutation rate ≥2% occurs frequently in both CLL and MM patients, the simultaneous occurrence of two different IGHV mutation rates ≥2% in either of these diseases is extremely rare. These findings strongly support that this case likely represents two independent clones rather than a single clonal evolution. Recently, next-generation DNA sequencing and/or tumor identification (ID) assays performed on liquid or solid biopsies have provided available genomic studies in the setting of concurrent hematological malignancies. Zhang et al. (5) identified high genomic similarity between malignant LN and BM samples via whole-genome sequencing, indicating a shared hematopoietic stem/progenitor cell origin for MM and CLL, and implicating copy number variation events as their potential drivers.

The clinical course of MM is usually more aggressive, whereas that of CLL is generally more indolent. Patients with concurrent MM and CLL commonly require treatment for MM and monitoring and close clinical follow-up without CLL treatment (6, 7). MM exhibits variability, and its treatment options include proteasome inhibitor, immunotherapy, corticosteroid, chemotherapy, and more recently, CD38-targeted monoclonal antibody. Radiotherapy alone is available to control local lesions of multiple solitary bone plasmacytoma. Of note, MFMM patients have better outcomes with novel treatments compared with typical symptomatic MM (3, 4, 13). Despite the coexistence of CLL, our patient responded well to myeloma-specific therapy, which was consistent with those previously reported in the literature. After three cycles of VRd, he achieved a complete response. The supraclavicular lymph nodes regressed to normal size, and the thoracic spine MRI showed a stable disease without new lesions. The patient’s clinical and laboratory parameters also showed improvement. Total leukocyte and absolute lymphocyte counts decreased from 57 × 10^9^/L to 8.5 × 10^9^/L and from 50.7 × 10^9^/L to 4.24 × 10^9^/L, respectively.

This case is unique, as it illustrates the rarity of coexisting MFMM and CLL. To our knowledge, this is the first report of concurrent CLL and MFMM presenting with EMP, characterized by low BM burden and high extramedullary/focal burden. Since lymphadenopathy is common among patients with CLL but rare in patients with MM, clinicians and radiologists are more likely to overlook the risk of EMP. Therefore, this case underscores the value of dual testing for plasma cell and B-cell populations if the clinical features are not entirely consistent (e.g., lymphocytosis with lytic bone lesions and lymphadenopathy, as in this case). When encountering similar cases of lymphocytosis with bone disease in the future, physicians should pay attention to the risk of CLL with MM to aviod missed diagnosis or misdiagnosis. Ancillary examinations such as morphological assessment, flow cytometry, pathological biopsy, molecular research, and PET-CT scan are indispensable in achieving this goal.

## Data Availability

The original contributions presented in the study are included in the article/**Supplementary Material**. Further inquiries can be directed to the corresponding author.
