# Supplementary figures and images for: Case Report: Simultaneous chronic lymphocytic leukemia and macrofocal multiple myeloma with extramedullary plasmacytoma
Source: Front Oncol. 2026 Jan 28;16:1747723. doi: 10.3389/fonc.2026.1747723 (PMC12890610; doi:10.3389/fonc.2026.1747723)

Supplementary Figure 1.


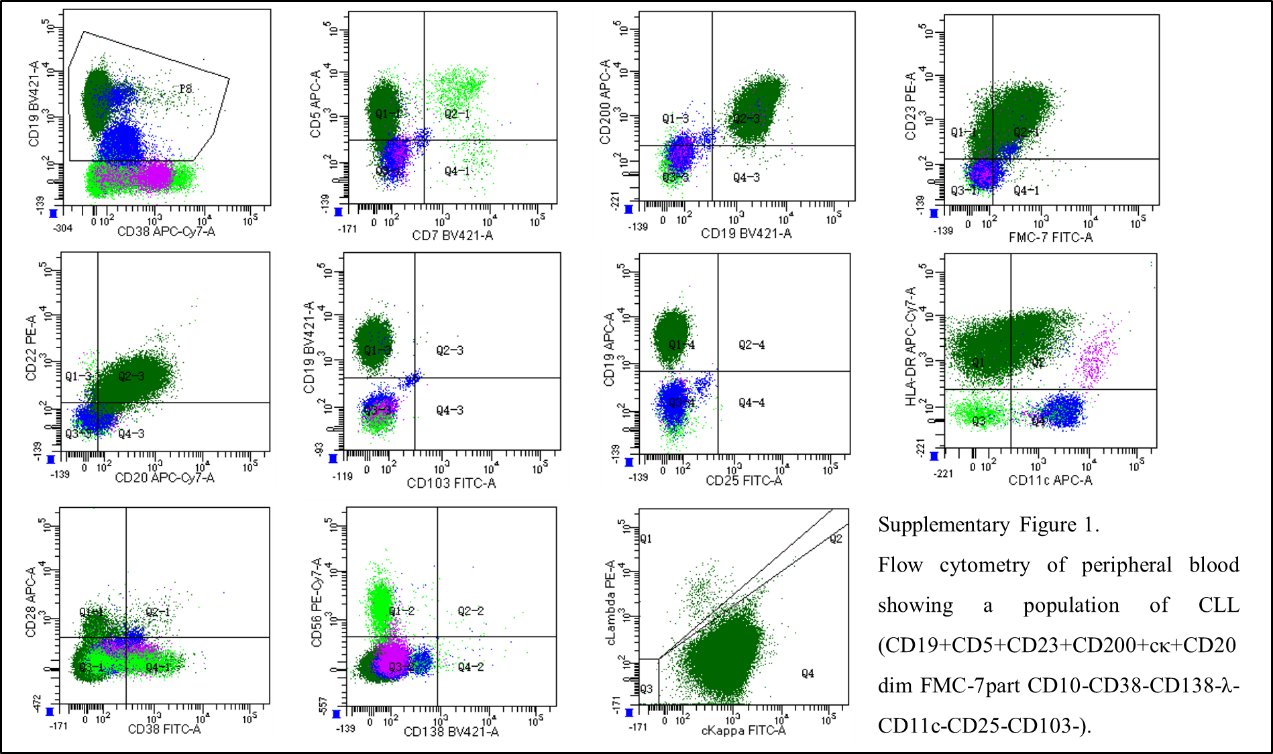

Supplement: Supplementary file 1 [file DataSheet1.docx]

Supplementary Figure 2.


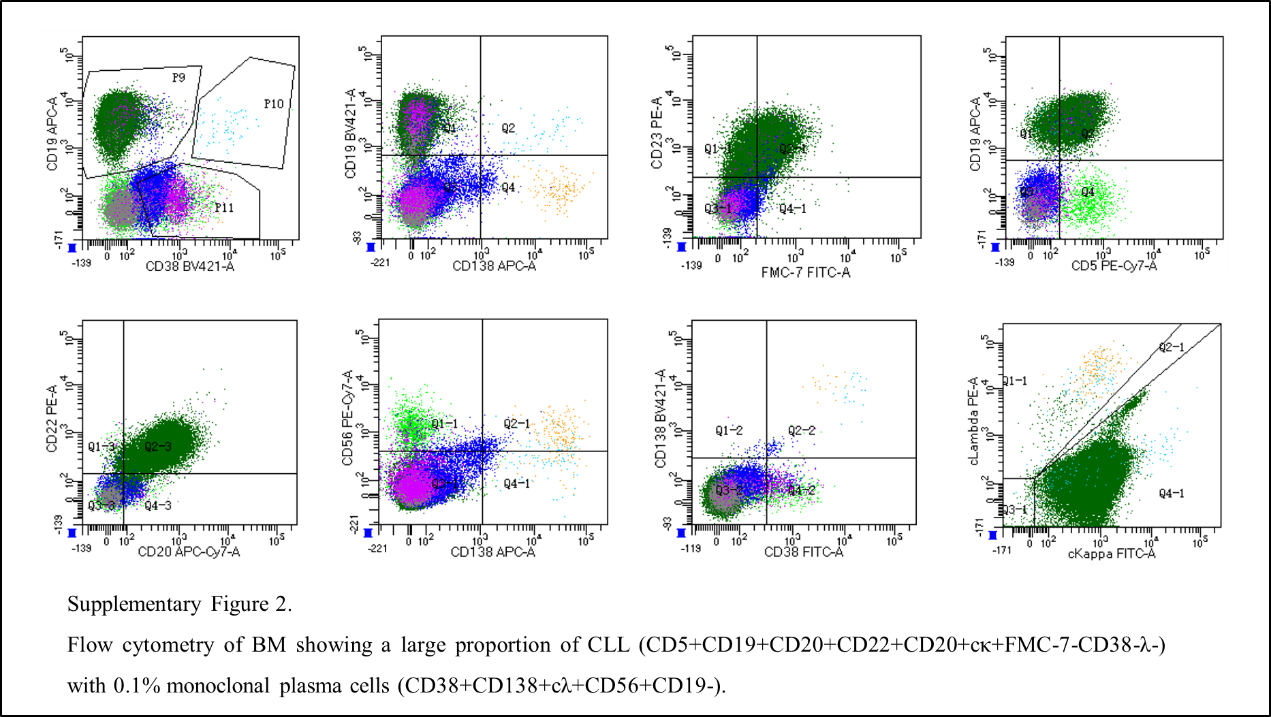

Supplement: Supplementary file 2 [file DataSheet2.docx]
