# Supplementary material for: Case Report: Simultaneous chronic lymphocytic leukemia and macrofocal multiple myeloma with extramedullary plasmacytoma
Source: Front Oncol. 2026 Jan 28;16:1747723. doi: 10.3389/fonc.2026.1747723 (PMC12890610; doi:10.3389/fonc.2026.1747723)
Supplement: Supplementary file 3 [file Presentation1.pptx]

## Slide 1
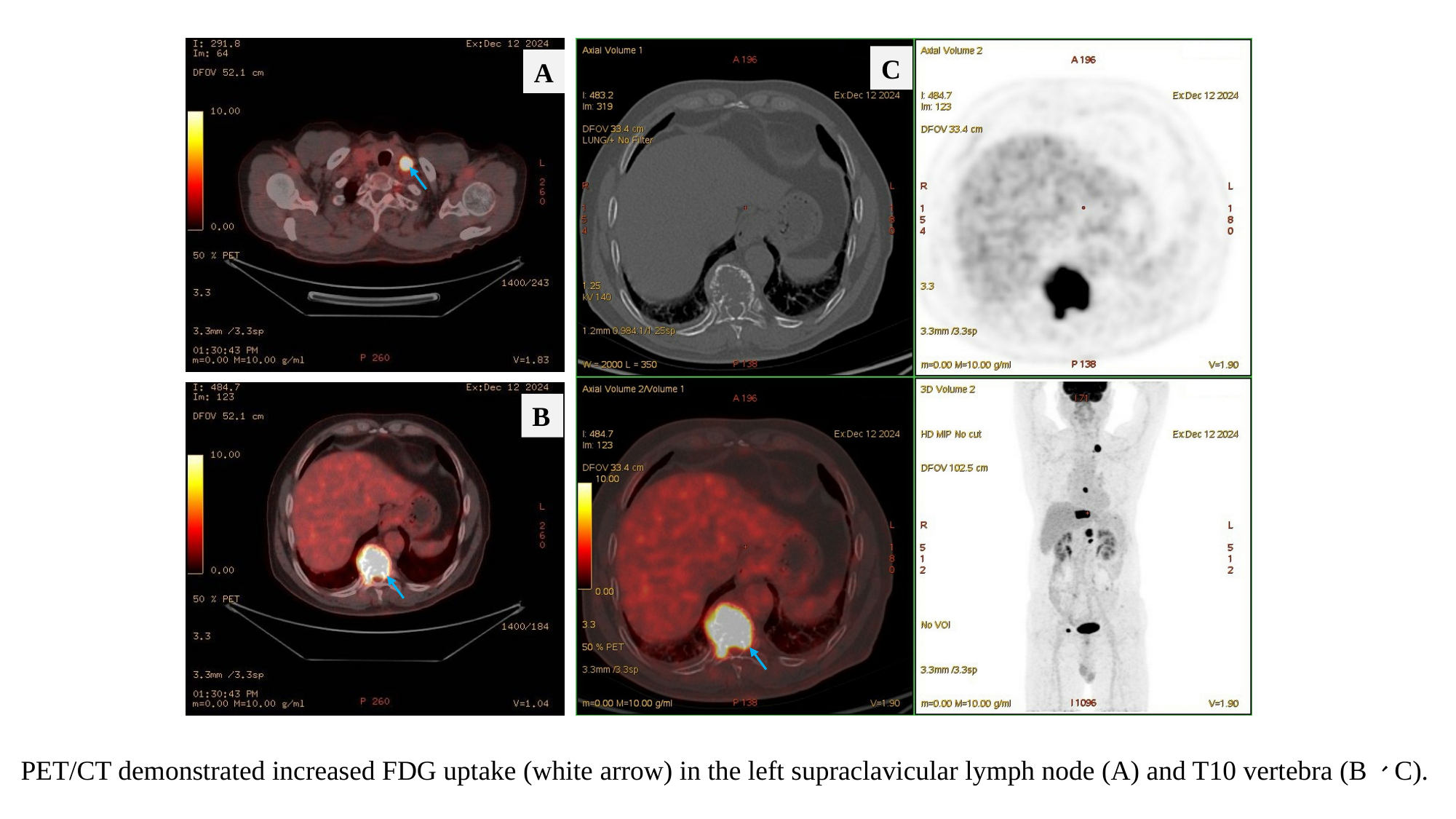

C
A
B
 PET/CT demonstrated increased FDG uptake (white arrow) in the left supraclavicular lymph node (A) and T10 vertebra (B、C).
